# Botulinum toxin type E alleviates trigeminal neuropathic pain via modulation of the HIF-1α–NLRP3 pathway

**DOI:** 10.3389/ftox.2026.1823460

**Published:** 2026-05-12

**Authors:** JoYoung Son, YuMi Kim, JinSook Ju, JaeYoung Kim, JeongSun Nam, YoonKyoung Cha, DongKuk Ahn

**Affiliations:** 1 Department of Oral Physiology, School of Dentistry, Kyungpook National University, Daegu, Republic of Korea; 2 JETEMA Global, Seongnam-si Gyeonggi-do, Republic of Korea

**Keywords:** antinociception, botulinum toxin type E, cytokines, HIF-1α, neuropathic pain, NLRP3

## Abstract

**Background:**

While botulinum neurotoxin type E (BoNT/E) has been shown to have analgesic effects in previous studies, the underlying mechanisms mediating its therapeutic effects remain incompletely understood. This study investigated the involvement of the nucleotide-binding oligomerization domain, leucine-rich repeat, and pyrin domain-containing protein 3 (NLRP3) inflammasome in the antinociceptive effects of BoNT/E in a rat model of trigeminal neuropathic pain.

**Methods:**

Neuropathic pain was induced in male Sprague–Dawley rats through inferior alveolar nerve (IAN) injury.

**Results:**

IAN injury significantly decreased air-puff thresholds, resulting in mechanical allodynia that persisted for up to 42 days. NLRP3 expression in the ipsilateral trigeminal ganglion (iTG) was significantly increased in the nerve-injured group on postoperative day (POD) 5. In contrast, subcutaneous administration of BoNT/E (6 or 10 U/kg) significantly attenuated mechanical allodynia and suppressed NLRP3 expression in the iTG on POD 5. Moreover, BoNT/E (10 U/kg) markedly reduced the elevated levels of inflammatory cytokines, including interleukin (IL)-1β, IL-18, tumor necrosis factor-alpha (TNF-α), and IL-6 in the iTG by IAN injury. The nerve-injured group also exhibited significant upregulation of hypoxia-inducible factor 1-alpha (HIF-1α) expression in the iTG, which was significantly decreased following BoNT/E (10 U/kg) treatment. Intraganglionic injection of PX-478, a HIF-1α inhibitor, similarly attenuated mechanical allodynia, downregulated NLRP3 expression, and decreased IL-1β, IL-18, TNF-α, and IL-6 levels in the iTG.

**Conclusion:**

Collectively, these findings demonstrate that modulation of the HIF-1α–NLRP3 pathway in the iTG plays a critical regulatory role in neuropathic pain development and suggest that BoNT/E may serve as a promising therapeutic strategy for managing chronic neuropathic pain.

## 1 Introduction

Neuropathic pain, characterized by primary lesions or dysfunction of the somatosensory system, often presents with peripheral symptoms such as burning or tingling sensations, as well as hypersensitivity to touch or cold (Baron, 2000; Woolf and Mannion, 1999). The causes of neuropathic pain are diverse and are often associated with underlying diseases or lesions that are difficult to manage (Baron, 2000; Woolf and Mannion, 1999). Unfortunately, neuropathic pain typically responds poorly to conventional analgesics (Cohen and Mao, 2014). As a consequence, despite the increasing availability of treatment options, effective management of neuropathic pain remains challenging for many patients. Therefore, there is an urgent and critical need for the development of novel therapeutic strategies capable of more effectively managing and controlling chronic neuropathic pain.

Botulinum neurotoxin type E (BoNT/E) is a major cause of human botulism, can induce severe symptoms including respiratory failure, muscle paralysis, and even death. Its molecular mechanisms are similar to those of botulinum neurotoxin type A (BoNT/A). Although BoNT/E cleaves synaptosome-associated protein 25 (SNAP-25) in a manner comparable to BoNT/A, it targets a distinct cleavage site, resulting in different protein fragments (Foran et al., 2002). Consequently, BoNT/E has a rapid onset of action and a markedly shorter duration of effect, with a biological half-life of fewer than 3 days, whereas BoNT/A exhibits a substantially longer half-life of 10–20 days (Lebowitz and Berson, 2022; Pons et al., 2019). Recently, BoNT/E has attracted increasing clinical interest because of these unique pharmacological properties. A randomized, double-blind, placebo-controlled clinical trial demonstrated that BoNT/E is both safe and highly effective for aesthetic treatment in patients with glabellar frown lines (Yoelin et al., 2018). Additionally, BoNT/E has been shown to exert long-lasting neuroprotective effects in experimental stroke models (Antonucci et al., 2010) and to display antiepileptogenic effects in preclinical epilepsy models (Costantin et al., 2005). More recently, its antinociceptive potential has also been highlighted (Jung et al., 2025). Subcutaneous administration of BoNT/E significantly attenuated nociceptive behaviors in the formalin test and reduced the complete Freund’s adjuvant (CFA)-induced thermal hyperalgesia (Jung et al., 2025). BoNT/E treatment also significantly attenuated neuropathic mechanical allodynia in an inferior alveolar nerve (IAN) injury model (Jung et al., 2025). Although accumulating evidence indicates that BoNT/E has therapeutic potential for chronic pain, the underlying mechanisms of its analgesic effects have not yet been fully elucidated.

The nucleotide-binding oligomerization domain, leucine-rich repeat, and pyrin domain-containing protein 3 (NLRP3) inflammasome is an intracellular sensor that plays a critical role in regulating innate immune responses and maintaining cellular homeostasis (Huang et al., 2021; Swanson et al., 2019). Recent evidence indicates that NLRP3 plays a key role in the development of chronic neuropathic pain. Upregulation of NLRP3 has been detected in both the sciatic nerve and dorsal root ganglia of rats with paclitaxel-induced neuropathy (Jia et al., 2017), and similar increases have been observed in the spinal cord following sciatic nerve injury (Xu et al., 2019). Intraganglionic injection of an NLRP3 inhibitor alleviated lysophosphatidic acid (LPA)-induced trigeminal neuralgia-like pain (Park et al., 2025), and suppression of spinal NLRP3 expression through miR-34c overexpression attenuated neuropathic pain in a chronic constrictive sciatic nerve injury model (Xu et al., 2019). Moreover, sciatic nerve ligation increased NLRP3 expression in the spinal cord, and intraperitoneal injection of MCC950, an NLRP3 inhibitor, suppressed NLRP3 expression and alleviated neuropathic pain (Shao et al., 2021). Taken together, these findings confirm the involvement of NLRP3 in the pathogenesis of chronic neuropathic pain following nerve injury. However, the mechanisms connecting NLRP3 activity and the antinociceptive effects of BoNT/E have not been explored.

The present study investigated the underlying mechanisms of BoNT/E-induced antinociception in a model of neuropathic pain. Specifically, we examined whether BoNT/E alleviates neuropathic mechanical allodynia in IAN-injured rats. We further evaluated the involvement of NLRP3 and inflammatory cytokines in the ipsilateral trigeminal ganglion (iTG). Additionally, we explored whether the hypoxia-inducible factor 1-alpha (HIF-1α) pathway contributes to the NLRP3-mediated antinociceptive effects of BoNT/E.

## 2 Materials and methods

### 2.1 Animals

All experimental protocols were approved by the Animal Experiment Ethics Committee of Kyungpook National University (Approval Code: KNU-2023-0368) and followed the ARRIVE 2.0 guidelines. A total of 268 adult male Sprague–Dawley rats (6–8 weeks; 220–240 g; 4 animals per cage) were obtained from the Central Lab for Animal Inc. (Seoul, Korea). The animals were housed in a pathogen-free environment under controlled room temperature (22 °C ± 2 °C), humidity (50%), and a light/dark cycle (12 h). The rats had *ad libitum* access to water and food. Prior to behavioral testing, all the rats were allowed to acclimate to the experimental environment for at least 30 min to minimize stress-related variability. All experimental procedures were executed under double-blind conditions to prevent observer bias during data acquisition. Neither the participants nor the investigators were aware of the group assignments. Group allocation was performed by an independent researcher. Additionally, rigorous protocols were implemented throughout the study to minimize the total number of animals used and to reduce pain and distress at every stage of the experimental process.

### 2.2 Animal models for trigeminal neuropathic pain

The IAN injury model was established based on previously reported protocols (Lee et al., 2019; Son et al., 2022). General anesthesia was induced by intramuscular injection of a ketamine (40 mg/kg) and xylazine (4 mg/kg) cocktail. The left mandibular second M was subsequently extracted, and a mini-dental implant (1 mm in diameter, 4 mm in length; MegaGen, Daegu, Korea) was inserted into the extracted socket to intentionally damage the IAN. Animals that underwent tooth extraction without implant insertion served as controls. For post-experimental evaluation, only data from rats in which implant placement was confirmed to have successfully induced IAN injury were included in the statistical analysis.

### 2.3 Evaluation of mechanical allodynia

Mechanical allodynia thresholds were assessed in a quiet room between 09:00 and 18:00. Pain-related behavioral responses were evaluated by delivering ten air-puff stimuli, each applied for 4 s with an interstimulus interval of 10 s, in accordance with previously described procedures (Kim et al., 2014; Lee et al., 2019; Kim et al., 2020). The intensity, duration, and interval of the air-puff stimuli were precisely controlled using a pneumatic pico-pump (World Precision Instruments, Sarasota, FL, United States). Stimulation was delivered at a 90° angle from a distance of 1 cm from the face using a 10-cm-long, 25-gauge metal tube, primarily targeting the whisker pad, corners of the mouth, and lower jaw. Mechanical allodynia was defined as an avoidance response to at least 50% of the stimuli. The air stimulus was administered with a cutoff pressure of 40 psi, as determined in previous literature (Han et al., 2012; Park et al., 2019). Notably, withdrawal responses were not observed in experimentally naïve rats at any pressure below this threshold.

### 2.4 Western blotting

Five days post-operation, iTG tissue (n = 6 per group) was immediately collected. The harvested tissues were frozen on dry ice and stored directly at −80 °C until the protein was analyzed. The iTG sample was homogenized according to established protocols (Cho et al., 2022; Jo et al., 2024). Western blotting was then performed to assess the protein expression levels of NLRP3 and HIF-1α. Total protein was extracted from the iTG samples by grinding followed by sonication in chilled lysis buffer supplemented with phosphatase and protease inhibitors (Thermo Fisher Scientific, Rockford, IL, United States). Sonication was carried out using a Bioruptor (Cosmo Bio, Tokyo, Japan). The lysates were then clarified by centrifugation at 13,000 rpm for 30 min. The protein concentrations were measured using a Qubit fluorometer system (Thermo Fisher, Scientific). Twenty micrograms of protein from each sample was loaded for separation by SDS–polyacrylamide gel electrophoresis (SDS–PAGE) using a NuPAGE 3%–8% gradient Tris-acetate gel (Invitrogen, Waltham, MA, United States), followed by transfer onto nitrocellulose membranes. After protein transfer, the membranes were blocked for 1 h at room temperature with 5% nonfat skim milk prepared in TBS-T buffer. The membranes were then incubated overnight at 4 °C with primary antibodies against NLRP3 (1:1000; Novus Biologicals, Centennial, CO, United States), HIF-1α (1:1000; Santa Cruz Biotechnology, Dallas, TX, United States), and the loading control GAPDH (glyceraldehyde-3-phosphate dehydrogenase; 1:10,000; Santa Cruz Biotechnology). The membranes were subsequently incubated with secondary anti-rabbit or anti-mouse IgG antibodies (1:5,000; Bio-Rad, Hercules, CA, United States). Immunoreactive bands were visualized using an enhanced chemiluminescence detection kit (Millipore, Billerica, MA, United States) and captured with an Amersham Imager 600 (GE Healthcare, Chicago, IL, United States). Band intensities were quantified using ImageJ (NIH, Bethesda, MD, United States), and relative expression levels were normalized to GAPDH.

### 2.5 Measurement of cytokine concentrations

The concentrations of cytokines, including interleukin (IL)-1β, IL-18, tumor necrosis factor-alpha (TNF-α), and IL-6, were measured in the iTG supernatant (*n* = 6 per group). Commercial enzyme-linked immunosorbent assay (ELISA) kits were used following the manufacturers’ instructions. ELISA kits for IL-1β, IL-6, and TNF-α were obtained from R&D Systems (Minneapolis, MN, United States), whereas the IL-18 kit was purchased from Boster Bio (Pleasanton, CA, United States). Absorbance was measured at 420–570 nm, and a standard curve was included in each experiment for quantitative analysis.

### 2.6 Chemicals

BoNT/E was provided by JETEMA Co., Ltd. (Wonju, Korea) at a concentration of 100 units per vial and reconstituted in 1 mL of sterile saline following a previously established protocol (Jung et al., 2025). PX-478 was obtained from Selleck Chemicals (Houston, TX, United States) and prepared by dissolving it in sterile saline.

### 2.7 Experimental protocols

#### 2.7.1 Changes in NLRP3 expression and cytokine concentrations in the iTG

IAN injury reliably induced significant mechanical allodynia. Mechanical allodynia was quantified by measuring air-puff thresholds at 13 different time points after injury (days 0, 2, 3, 7, 10, 14, 17, 21, 24, 28, 35, 42, and 50; *n* = 7 per group). Changes in NLRP3 expression in the iTG following IAN injury were evaluated using Western blot analysis. Additionally, the concentrations of proinflammatory cytokines (IL-1β, IL-18, TNF-α, and IL-6) in the iTG were measured using ELISA. Tissue sampling for these biochemical analyses (*n* = 6 per group) was performed 5 days after IAN injury.

#### 2.7.2 Effects of BoNT/E on neuropathic pain

The effects of BoNT/E on mechanical allodynia induced by IAN injury were evaluated. As expected, IAN injury produced robust neuropathic mechanical allodynia. BoNT/E was administered subcutaneously on postoperative day (POD) 3 at doses of 2, 6, and 10 U/100 µL/kg. The injection site corresponded to the area most responsive to air-puff stimulation. Therapeutic efficacy was evaluated by measuring air-puff thresholds at multiple time points (days 0, 3, 4, 5, 6, 7, 9, 11, 14, 18, 21, 25, 32, 40, and 50) after BoNT/E or vehicle administration (*n* = 7 per group).

#### 2.7.3 Changes in NLRP3 expression and cytokine levels following BoNT/E injection

The involvement of the NLRP3-cytokine pathway in the pain-relieving effects of BoNT/E was also investigated. Three days after IAN injury, BoNT/E (10 U/100 µL/kg) was administered subcutaneously. Changes in NLRP3 expression were assessed by Western blotting, and the concentrations of the proinflammatory cytokines IL-1β, IL-18, TNF-α, and IL-6 were determined by ELISA. Tissue samples from the iTG (*n* = 6 per group) were collected 5 days after IAN injury for all molecular analyses.

#### 2.7.4 Changes in HIF-1α expression in the iTG following BoNT/E treatment

HIF-1α expression in the iTG following nerve injury was evaluated, and changes after BoNT/E treatment were also examined. A single dose of BoNT/E (10 U/100 µL/kg) was administered 3 days after nerve injury. HIF-1α expression was quantified using Western blot analysis. Protein samples for this analysis (*n* = 6 per group) were collected from the iTG 5 days after nerve injury.

#### 2.7.5 Effects of HIF-1α pathway inhibition on neuropathic mechanical allodynia, NLRP3 expression, and cytokine concentrations

To investigate the functional role of HIF-1α upregulation following nerve injury, a guide cannula was surgically implanted into the iTG on the day of IAN injury using an established technique (Ahn et al., 2005; Han et al., 2012). The HIF-1α inhibitor PX-478, which suppresses HIF-1α transcription via thioredoxin-1 inhibition (Koh et al., 2015; Samaranayake et al., 2017), was intraganglionically injected (0.5 and 1 µg) under isoflurane anesthesia on POD 5. Air-puff thresholds were measured at multiple time points (hours 0, 0.5, 1, 1.5, 2, 2.5, 3, 3.5, 4, 5, 6, and 24) following PX-478 administration (*n* = 7 per group). In addition, changes in NLRP3 expression and the concentrations of IL-1β, IL-18, and TNF-α in the iTG were evaluated after HIF-1α pathway inhibition. Protein samples for these biochemical analyses (*n* = 6 per group) were collected 5 hours after PX-478 injection.

### 2.8 Data analysis

Statistical analyses were performed using SPSS (version 30.0). Nociceptive behavioral data collected over time were analyzed using repeated-measures analysis of variance (ANOVA), followed by the Holm–Sidak *post hoc* test. For single time-point comparisons among multiple groups, one-way ANOVA was performed, followed by the Holm–Sidak *post hoc* test. Comparisons between two groups were performed using Student’s t*-*test. A *p* value of <0.05 was considered to indicate statistical significance. All the data are presented as the mean ± standard error of the mean (SEM).

## 3 Results

### 3.1 Changes in NLRP3 levels in the iTG following IAN injury

**FIGURE 1:**
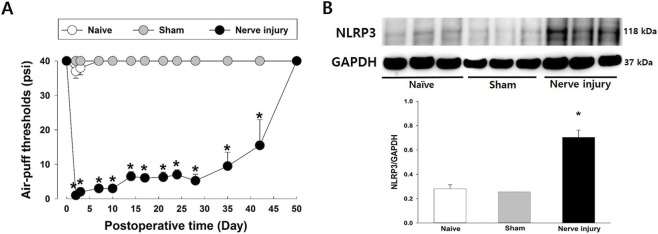
Alterations in air puff thresholds and the nucleotide-binding oligomerization domain, leucine-rich repeat, and pyrin domain-containing protein 3 (NLRP3) expression in the ipsilateral trigeminal ganglion (iTG) following inferior alveolar nerve (IAN) injury. **(A)** IAN injury significantly reduced air-puff thresholds, indicating the development of mechanical allodynia. This neuropathic state persisted for up to 42 days post-injury. Each group included seven animals. The data were presented as mean values with standard error of the mean (±SEM) and subjected to repeated measures analysis of variance (ANOVA) followed by Holm-Sidak *post hoc* tests. *Symbol show the significance between the sham and IAN injury groups (*p* < 0.05). **(B)** Changes in NLRP3 expression in the iTG were assessed by Western blot analysis. Compared with the sham treatment group, IAN injury significantly increased NLRP3 expression on POD 5. GAPDH served as the loading control. Each group included six animals. The data were presented as mean values with SEM, followed by one-way ANOVA with Holm-Sidak *post hoc* tests. *Symbol show the significance between the sham and IAN injury groups (*p* < 0.05).

Changes in air-puff thresholds and NLRP3 expression in the iTG following IAN injury are shown in Figure 1. The naïve and sham-treated groups maintained stable air-puff thresholds throughout the experimental observation period. In contrast, rats subjected to IAN injury showed a significant reduction in air-puff thresholds, indicative of robust mechanical allodynia (F_(2,18)_ = 244.498; *P* < 0.05; Figure 1A). This decrease was observed from POD 2 and persisted for more than 42 days. Consistent with the behavioral changes, analysis of the iTG on POD 5 revealed significantly increased NLRP3 expression in the nerve-injured group compared with both the naïve and sham-treated groups (*P* < 0.05; Figure 1B).

### 3.2 Effects of BoNT/E on mechanical allodynia and NLRP3 expression

**FIGURE 2:**
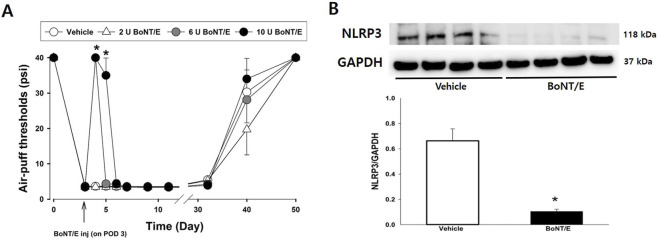
Changes in air puff thresholds and NLRP3 expression following subcutaneous botulinum toxin type E (BoNT/E) administration in IAN-injured rats. **(A)** Vehicle treatment and a low dose of BoNT/E (2 U/kg) did not alter air-puff thresholds. In contrast, BoNT/E at doses of 6 and 10 U/kg produced significant antiallodynic effects compared with the vehicle-treated group. The analgesic effect of the 10 U/kg dose was sustained for up to 48 h. Each group included seven animals. The data were presented as mean values with ±SEM, followed by repeated measures ANOVA with Holm-Sidak *post hoc* tests. *Symbol show the significance between the Vehicle and BoNT/E-treated groups (*p* < 0.05). **(B)** Western blot analysis revealed that BoNT/E (10 U/kg) significantly inhibited the IAN injury-induced upregulation of NLRP3 expression in the iTG compared with the vehicle-treated group. GAPDH served as the loading control. Each group included six animals. The data were presented as mean values with SEM, followed by by Student’s t*-*test. *Symbol show the significance between the vehicle and BoNT/E (10 U/kg)-treated groups (*p* < 0.05).

The effects of BoNT/E treatment on air-puff thresholds and NLRP3 expression are shown in Figure 2. BoNT/E was administered 3 days after nerve injury. Although the 2 U/kg dose did not alter air-puff thresholds, higher doses (6 and 10 U/kg) significantly alleviated mechanical allodynia (F_(3,24)_ = 1888.9; *P* < 0.05; Figure 2A). In our previous study, we observed a maximal antiallodynic effect 8 h after administration of 10 U/kg BoNT/E, with recovery occurring approximately 48 h later (Jung et al., 2025). In the present study, BoNT/E treatment (10 U/kg) produced anti-allodynic effects for 48 h after injection. However, BoNT/E treatment did not significantly affect the long-term recovery period in IAN-injured rats which extended beyond POD 50. Consistent with the behavioral findings, analysis of NLRP3 expression in the iTG on POD 5 revealed that treatment with 10 U/kg BoNT/E significantly reduced NLRP3 expression compared with the vehicle-treated group (*P* < 0.05; Figure 2B).

### 3.3 Changes in cytokine concentrations in the iTG following BoNT/E treatment

**FIGURE 3:**
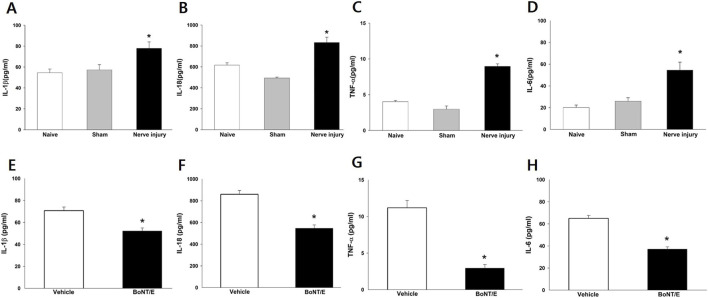
Changes in the concentrations of proinflammatory cytokines (interleukin [IL]-1β, IL-18, tumor necrosis factor [TNF]-α, and IL-6) in the iTG. **(A–D)** Compared with the sham group, IAN-injured rats exhibited significantly increased concentrations of IL-1β, IL-18, TNF-α, and IL-6 in the iTG. Each group included six animals. The data were presented as mean values with SEM, followed by one-way ANOVA with Holm-Sidak *post hoc* tests. *Symbol show the significance between the sham and IAN injury groups (*p* < 0.05). **(E–H)** Treatment with BoNT/E (10 U/kg) significantly reduced the elevated concentrations of IL-1β, IL-18, TNF-α, and IL-6 in the iTG. Each group included six animals. The data were presented as mean values with SEM, followed by Student’s t test. *Symbol show the significance between the vehicle and BoNT/E (10 U/kg)-treated groups (*p* < 0.05).

The cytokine responses in the iTG following BoNT/E treatment are shown in Figure 3. ELISA analysis revealed no significant differences in the concentrations of IL-1β, IL-18, TNF-α, or IL-6 between the naïve and sham control groups. In contrast, IAN injury significantly increased the concentrations of all four cytokines (*P* < 0.05; Figures 3A–D). Administration of 10 U/kg BoNT/E significantly attenuated the elevated levels of IAN injury-induced IL-1β, IL-18, TNF-α, and IL-6 (*P* < 0.05; Figures 3E–H).

### 3.4 HIF-1α expression in the iTG following BoNT/E treatment

**FIGURE 4:**
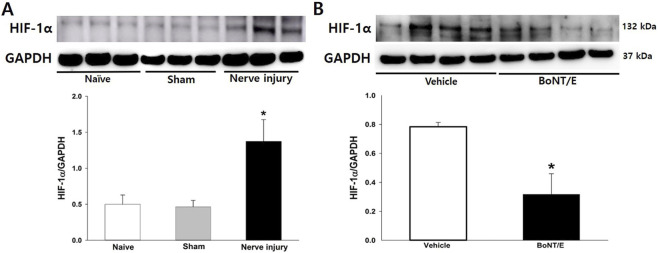
Changes in HIF-1α expression following BoNT/E injection. **(A)** Western blot analysis showed that IAN injury significantly increased HIF-1α expression in the iTG on POD 5 compared with the sham group. Each group included six animals. The data were presented as mean values with ±SEM, followed by one-way ANOVA with Holm-Sidak *post hoc* tests. *Symbol show the significance between the sham and IAN injury groups (*p* < 0.05). **(B)** Compared with vehicle treatment, BoNT/E injection significantly reduced the IAN injury-induced upregulation of HIF-1α expression on POD 5. Each group included six animals. The data were presented as mean values with SEM, followed by Student’s t*-*test. *Symbol show the significance between the vehicle and BoNT/E (10 U/kg)-treated groups (*p* < 0.05).

As shown in Figure 4A, HIF-1α expression in the iTG was significantly upregulated on POD 5 following IAN injury, whereas no change was observed in the sham group (*P* < 0.05). BoNT/E was administered on POD 3, and HIF-1α levels were assessed 2 days later by Western blot analysis. Treatment with 10 U/kg BoNT/E significantly reduced the IAN injury-induced increase in HIF-1α expression in the iTG (*P* < 0.05; Figure 4B).

### 3.5 Role of the HIF-1α pathway in the antinociceptive effects of NLRP3

**FIGURE 5:**
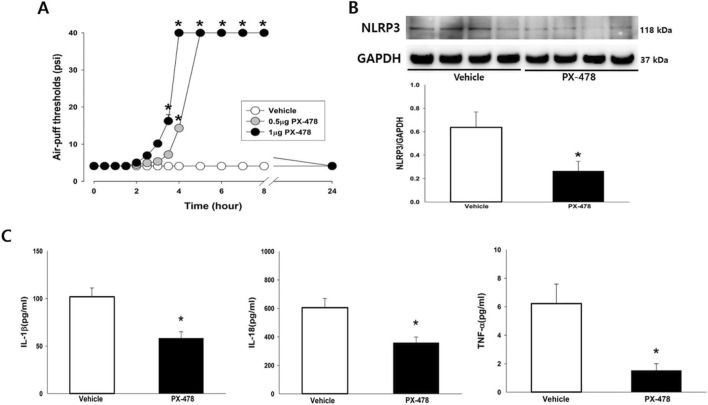
Involvement of the HIF-1α pathway in trigeminal neuropathic pain following IAN injury. **(A)** Effects of pharmacological blockade of the HIF-1α pathway on mechanical allodynia. On POD 5, intraganglionic administration of PX-478 (0.5 µg, 1 µg), a HIF-1α inhibitor, significantly alleviated mechanical allodynia. The antiallodynic effects peaked at 3 h and persisted until 8 h post-injection. Withdrawal thresholds returned to pretreatment values after 24 h. Each group included seven animals. The data were presented as mean values with SEM, followed by repeated measures ANOVA with Holm-Sidak *post hoc* tests. * Symbol show the significance between the vehicle and PX-478-treated groups (*p* < 0.05). **(B)** Effects of HIF-1α pathway blockade on NLRP3 expression. Intraganglionic injection of PX-478 significantly reduced NLRP3 expression in the iTG compared with the vehicle-treated group. Each group included six animals. The data were presented as mean values with SEM, followed by Student’s t*-*test. *Symbol show the significance between the vehicle and PX-478-treated groups (*p* < 0.05). **(C)** Effects of HIF-1α pathway blockade on cytokine concentrations. PX-478 administration significantly attenuated the elevated levels of IL-1β, IL-18, and TNF-α in the iTG. Each group included six animals. The data were presented as mean values with SEM, followed by Student’s t*-*test. *Symbol show the significance between the vehicle and PX-478-treated groups (*p* < 0.05).

The effects of HIF-1α pathway inhibition on mechanical allodynia, NLRP3 expression, and cytokine concentrations were investigated. Intraganglionic injection of PX-478 (0.5 µg and 1 µg), a HIF-1α inhibitor, significantly attenuated mechanical allodynia at 3 h after injection (F_(2,16)_ = 614.20; *P* < 0.05; Figure 5A). The antiallodynic effects persisted for up to 8 h and returned to pre-treatment levels by 24 h. In addition, intraganglionic injection of 1 µg PX-478 significantly reduced NLRP3 expression in the iTG (*P* < 0.05; Figure 5B). The elevated levels of IL-1β, IL-18, and TNF-α induced by IAN injury were also significantly decreased following PX-478 administration compared with vehicle treatment (*P* < 0.05; Figure 5C).

## 4 Discussion

This study provides the first evidence implicating the HIF-1α–NLRP3 axis in the pathogenesis of trigeminal neuropathic pain. IAN injury markedly increased both HIF-1α and NLRP3 expression in the iTG. Treatment with BoNT/E markedly attenuated mechanical allodynia and simultaneously suppressed the injury-induced upregulation of HIF-1α and NLRP3. BoNT/E also effectively reduced the elevated levels of proinflammatory cytokines, including IL-1β, IL-18, TNF-α, and IL-6. Importantly, direct pharmacological inhibition of the HIF-1α pathway also produced robust antiallodynic effects, accompanied by reduced NLRP3 expression and decreased cytokine production. Taken together, these findings strongly indicate that the HIF-1α–NLRP3 signaling within the iTG plays a crucial role in the development of trigeminal neuropathic pain following nerve injury and suggest that BoNT/E may represent a promising therapeutic agent for managing chronic neuropathic pain.

### 4.1 Antinociceptive effects of BoNT/E

BoNT, a potent neurotoxic protein produced by the anaerobic bacterium *Clostridium botulinum*, exerts its primary biological effect by inhibiting acetylcholine release at peripheral motor nerve terminals (Montecucco and Molgó, 2005; de Paiva et al., 1993; Rizo and Südhof, 1998). Similar to BoNT/A, BoNT/E cleaves SNAP-25, thereby inhibiting synaptic vesicle fusion and neurotransmitter release (Agarwal and Swaminathan, 2008; Jones et al., 2008; Ko et al., 2014). Despite these shared molecular mechanisms, relatively few studies have investigated the role of BoNT/E in pain modulation. The present study fills this gap by showing that IAN injury produced by a malpositioned dental implant induces pronounced mechanical allodynia, which is significantly alleviated by subcutaneous administration of BoNT/E in this well-established animal model. Previous studies have established BoNT as a promising therapeutic agent for chronic pain. For example, BoNT/A alleviates neuropathic pain associated with sciatic nerve injury (Bach-Rojecky et al., 2005; Luvisetto et al., 2007), spinal nerve ligation (Park et al., 2006), and IAN injury (Yang et al., 2016). More recent behavioral findings further support the analgesic potential of BoNT/E, demonstrating its ability to reduce inflammatory pain and mechanical allodynia following IAN injury (Jung et al., 2025), accompanied by decreased *c-fos* expression in the trigeminal subnucleus caudalis. Collectively, these findings identify BoNT/E as a promising therapeutic candidate for chronic pain conditions. However, the precise molecular mechanisms underlying its antinociceptive actions remain incompletely understood and require further investigation.

### 4.2 Role of NLRP3 and cytokines in BoNT/E-induced antinociceptive effects

The present study demonstrated that BoNT/E markedly suppressed the IAN injury-induced upregulation of NLRP3 in the iTG. Previous studies have strongly implicated NLRP3 in the pathogenesis of neuropathic pain, with increased expression observed in the dorsal root ganglia and sciatic nerve following paclitaxel treatment (Jia et al., 2017), as well as in the spinal cord after sciatic nerve injury (Xu et al., 2019). Pharmacological inhibition of the NLRP3 pathway alleviates neuropathic pain in chronic constrictive sciatic nerve injury models (Xu et al., 2019) and following sciatic nerve ligation (Shao et al., 2021). Consistent with these findings, NLRP3 knockout mice exhibit reduced pain sensitivity after sciatic nerve injury (Cui et al., 2020), collectively confirming the critical involvement of NLRP3 in the development of chronic neuropathic pain. Importantly, our results provide the first direct evidence that NLRP3 contributes to the analgesic mechanism of BoNT/E. BoNT/E treatment significantly inhibited the IAN injury-induced upregulation of NLRP3 expression in iTG. Furthermore, IAN injury markedly increased the concentrations of IL-1β, IL-18, TNF-α, and IL-6, all of which were subsequently reduced following BoNT/E administration. These findings indicate that BoNT/E modulates both NLRP3 expression and its associated downstream cytokine responses within the iTG. The mechanistic link between NLRP3 activation and cytokine release is well established, as the activation of NLRP3 leads to the activation of inflammatory caspases, resulting in the maturation and release of key cytokines such as IL-1β and IL-18 (Chen and Smith, 2023; Huang et al., 2021; Jia et al., 2017; Jo et al., 2016). Supporting this relationship, intraganglionic administration of an NLRP3 inhibitor was previously shown to block mechanical allodynia and significantly reduce the elevated levels of IL-1β, IL-18, and TNF-α in the iTG of LPA-injected rats (Park et al., 2025). Collectively, our findings demonstrate that BoNT/E suppresses both NLRP3 activation and the downstream cytokine production, strongly supporting the conclusion that BoNT/E exerts its antinociceptive effects, at least partially, through modulation of the NLRP3-cytokine signaling pathway in the iTG.

### 4.3 Role of HIF-1α in the antinociceptive effects of BoNT/E

HIF-1α is an oxygen-dependent transcriptional activator that plays a critical role in coordinating cellular responses to hypoxia, including tissue protection, wound healing, cell proliferation, and angiogenesis (Charpentier et al., 2016; Lee et al., 2004). Previous studies have reported HIF-1α in chronic inflammatory pain (Wang et al., 2025), and its expression has been shown to increase following sciatic nerve injury, with laser therapy-induced pain relief reducing its upregulation (Hsieh et al., 2012). Although these findings suggest a role for HIF-1α in chronic pain, it has remained unclear whether the HIF-1α–NLRP3 pathway specifically contributes to neuropathic pain after nerve injury. The present study addressed this gap by demonstrating that pharmacological inhibition of the HIF-1α significantly alleviates mechanical allodynia and suppresses NLRP3 expression in the iTG following IAN injury. In addition, inhibition of HIF-1α significantly reduced the IAN injury-induced elevation of IL-1β, IL-18, TNF-α, and IL-6 in the iTG compared with vehicle treatment. A previous study demonstrated that intraganglionic administration of an NLRP3 inhibitor blocked mechanical allodynia and reduced the elevated levels of IL-1β, IL-18, and TNF-α in the iTG (Park et al., 2025). These findings strongly indicate that the HIF-1α-NLRP3 pathway acts as a key molecular mediator in the development of neuropathic pain following nerve injury. Importantly, IAN injury markedly upregulated HIF-1α and NLRP3 expression, whereas BoNT/E administration significantly suppressed these increases. Collectively, these findings suggest that the antinociceptive effects of BoNT/E are mediated, at least in part, through modulation of the HIF-1α–NLRP3 pathway in the iTG. Therefore, targeting this HIF-1α–NLRP3 axis represents a novel and potentially effective therapeutic strategy for neuropathic pain.

Although the present data showed that intraganglionic injection of PX-478, a HIF-1α inhibitor, significantly reduced NLRP3 expression in the iTG, it remains unclear whether HIF-1α directly binds to the NLRP3 promoter or regulates NLRP3 expression indirectly through oxidative stress. Previous studies have reported that HIF-1α enhances the expression of phosphorylated NLRP3 by recruiting caspase-1 in chronic rhinosinusitis (Zhong et al., 2023). Moreover, HIF-1α has been shown to act as a positive regulator of NLRP3 inflammasome activation via NF-κB signaling in hypoxic microenvironments and pulpal inflammation (Wang et al., 2024). Moreover, previous studies have demonstrated the cellular localization of HIF-1α within satellite glial cells in the trigeminal ganglion in an animal model of medication-overuse headache (Topa et al., 2025). Double immunofluorescence staining has also revealed that NLRP3 colocalizes with the microglial marker in the trigeminal ganglion in a migraine animal model (He et al., 2019). These findings suggest that NLRP3 and HIF-1α, which are expressed in the trigeminal ganglion following nerve injury, may contribute to the development of neuropathic pain through complex cellular interactions. Therefore, further studies are required to elucidate the precise underlying mechanisms.

Importantly, IAN injury markedly upregulated HIF-1α expression, whereas BoNT/E administration significantly suppressed this increase. Collectively, these findings suggest that the antinociceptive effects of BoNT/E are mediated, at least in part, through modulation of the HIF-1α–NLRP3 pathway in the iTG. Therefore, targeting this HIF-1α–NLRP3 axis represents a novel and potentially effective therapeutic strategy for neuropathic pain.

### 4.4 Limitation and clinical perspectives

Several limitations of the present study warrant consideration. Although the IAN injury model effectively induces robust nociceptive behaviors in rats, it may not fully encapsulate the multifaceted pathophysiology characterizing human trigeminal neuropathic pain. This inherent biological discrepancy potentially restricts the direct translation of these findings into clinical settings. Furthermore, in light of established sexual dimorphism in pain processing (Dao and LeResche, 2000) and the modulatory influence of sex hormones on nociceptive thresholds (Bartley and Fillingim, 2013; Osborne and Davis, 2022), this study was limited to male Sprague-Dawley rats to eliminate the confounding variability of hormonal cycles. Consequently, these findings may not be generalizable to female populations. Nevertheless, the potent antinociceptive efficacy of BoNT/E demonstrated here provides a significant mechanistic foundation for developing novel therapies for chronic neuropathic pain.

In the present study, no overt signs of systemic toxicity or motor impairment, such as reduced mobility, abnormal posture, or feeding difficulties, were observed following administration of BoNT/E at doses up to 10 U/kg. The animals appeared behaviorally normal throughout the observation period. Consequently, the doses of 6 and 10 U/kg used in this study were considered appropriate, as they produced significant analgesic effects in experimental animals without observable functional impairment. Taken together, these findings suggest that BoNT/E provides prolonged analgesic effects and may represent a promising therapeutic option for the management of neuropathic pain.

BoNT/A and BoNT/E differ in their duration of action. BoNT/A produces long-lasting effects that can persist for several weeks to months, whereas BoNT/E induces a more rapid but short-lived effect, typically lasting only days. This difference is primarily attributed to their distinct SNARE protein targets and intracellular stability. Clinically, BoNT/E may be advantageous in conditions requiring rapid onset and transient effects, while BoNT/A is preferred for sustained therapeutic outcomes (Rossetto et al., 2014; Eleopra et al., 2020; Pirazzini et al., 2017).

## 5 Conclusion

In summary, subcutaneous BoNT/E treatment significantly alleviated mechanical allodynia and suppressed the IAN injury-induced upregulation of NLRP3 and HIF-1α expression in the iTG. BoNT/E also reduced the elevated concentrations of IL-1β, IL-18, TNF-α, and IL-6 in the iTG. Furthermore, the antiallodynic effects, reduction in NLRP3 expression, and decreases in cytokine levels observed following pharmacological blockade of the HIF-1α pathway confirm its critical role in the neuropathic pain cascade. Because BoNT/E directly suppresses the injury-induced HIF-1α upregulation, these findings strongly support the conclusion that modulation of the HIF-1α–NLRP3 pathway in the iTG underlines the development of trigeminal neuropathic pain. Collectively, these findings identify BoNT/E as a promising therapeutic strategy for treatment of chronic neuropathic pain.

## Data Availability

The original contributions presented in the study are included in the article/supplementary material, further inquiries can be directed to the corresponding author.
